# Strain-Associated Variations in Abnormal PrP Trafficking of Sheep Scrapie

**DOI:** 10.1111/j.1750-3639.2008.00150.x

**Published:** 2009-01

**Authors:** Martin Jeffrey, Gillian McGovern, Caroline M Goodsir, Silvia Síso, Lorenzo González

**Affiliations:** Veterinary Laboratories Agency (Lasswade), Pentlands Science Park, Bush Loan, Penicuik, MidlothianEdinburgh, UK

**Keywords:** endocytosis, prion disease, scrapie, stroin

## Abstract

Prion diseases are associated with the accumulation of an abnormal form of the host-coded prion protein (PrP). It is postulated that different tertiary or quaternary structures of infectious PrP provide the information necessary to code for strain properties. We show here that different light microscopic types of abnormal PrP (PrP^d^) accumulation found in each of 10 sheep scrapie cases correspond ultrastructurally with abnormal endocytosis, increased endo-lysosomes, microfolding of plasma membranes, extracellular PrP^d^ release and intercellular PrP^d^ transfer of neurons and/or glia. The same accumulation patterns of PrP^d^ and associated subcellular lesions were present in each of two scrapie strains present, but they were present in different proportions. The observations suggest that different trafficking pathways of PrP^d^ are influenced by strain and cell type and that a single prion strain causes several PrP^d^–protein interactions at the cell membrane. These results imply that strains may contain or result in production of multiple isoforms of PrP^d^.

## INTRODUCTION

Scrapie of sheep is the prototype of a group of fatal neurological diseases called transmissible spongiform encephalopathies (TSEs) or prion diseases that include bovine spongiform encephalopathy (BSE) of cattle and Creutzfeldt–Jakob disease of man. The cause of the TSEs or prion diseases is still a matter of some debate, but most investigators now favor the prion hypothesis (40, 41), which postulates that the cause of prion diseases is an abnormal form of a host-coded, cell surface, sialoglycoprotein called prion protein (PrP). For many years, a major challenge to the prion hypothesis has been to explain how an abnormal protein could code for the existence of numerous, stable, well-characterized rodent scrapie strains. According to this hypothesis, strain-specific information is encoded within the conformation of different tertiary or quaternary structures of an infectious isoform of PrP (41, 46).

Murine scrapie strains are characterized according to their relative incubation periods in different inbred mouse lines, different patterns of pathology and immunochemical forms of abnormal PrP accumulation (3, 43). A smaller number of experimental sheep scrapie strains have been recognized by their relative incubation times in different PrP genotypes (18). Biochemical methods are not readily able to distinguish between natural scrapie sources, although two experimental strains, CH1641 and BSE, have distinctive molecular phenotypes (17, 26); however, both experimental and naturally occurring scrapie sources may be distinguished by pathological phenotypes (14). The specific parameters of the pathological phenotype that permit differentiation of strains differs for sheep and mice: different murine strains show marked variation in vacuolation and neuroanatomic targeting (4), but sheep scrapie strains and sources mainly affect the same brain areas and neurons (14). Following immunohistochemical labeling, individual sheep strains and sources show highly diverse morphological types of disease-associated PrP (PrP^d^) accumulation (14), whereas cloned murine strains have fewer types of PrP^d^ accumulation. Although the PrP genotype may affect the magnitude of PrP^d^ accumulation in sheep, the patterns and types of PrP^d^ accumulation are not influenced by breed or route of inoculation (15, 35). From studies of the proportions of different types of PrP^d^ accumulation (the PrP^d^ profile), we have proposed that the pathology phenotype of sheep strains are partly the result of differences in the relative affinity of different sheep scrapie agents for different cell types and by differences in the cellular processing of PrP^d^(15). In the present study, we have sought to determine the nature of these putative processing or trafficking pathways of PrP^d^ in different sheep scrapie sources.

In previous electron microscopy immunogold studies of rodent scrapie, we have shown that PrP^d^ accumulates in the extracellular space and at the plasma membrane of neurons, glial cells and follicular dendritic cells (21, 25, 37). The degree to which the plasma membrane and extracellular forms show a tendency to aggregate and form fibrils differs between strains and tissues, but no differences in the nature of the changes in neurons of different brain areas were found. In contrast, variation in neuronal pathology both within and between neuroanatomic sites may be found for different sheep scrapie sources (7, 8). To determine whether there are different processing pathways for PrP^d^ sheep scrapie strains, we first determined the *in vivo* sites of accumulation and processing of PrP^d^ in different naturally occurring sheep scrapie sources and then investigated possible molecular mechanisms for some of these changes in brain and in lymphoid tissues (the latter is reported separately in reference (36). Our results suggest that varying proportions of several functional conformers are present in the brains of different sheep scrapie strains.

## MATERIALS AND METHODS

The sheep included in the study and the methods of tissue fixation have been described previously (7). Briefly, ten clinically affected sheep with natural scrapie and four uninfected control animals from New Zealand were perfused under deep surgical anaesthesia with mixed aldehydes and post-fixed in osmium tetroxide. PrP genotypes and farms of origin are listed in Table 1. The polymorphisms at codons 136, 154 and 171 in the ovine PrP gene are indicated with the appropriate amino acid single-letter code.

**Table 1:** Sheep genotypes, farm of origin and presence or absence of selected light and electron microscopy lesions. Abbreviation: PrP = prion protein; WMAS = white matter astrocyte associated PrP^d^.

Sheep genotype	Farm	Light microscopy			Electron microscopy*					
		Vascular plaques	Stellate	WMAS	PrP^d^ + ve neuronal/dendrites	PrP^d^ + ve pits and membrane changes	spiral inclusions (PrP^d^ + or − ve)	PrP^d^ + ve astrocytic membrane proliferation	PrP^d^ + ve lysosomes	PrP^d^ − ve reactive astrocytosis
R881 ARQ/ARQ	A	−	+++	++	+++	+++	+	+	++	+++
R883 ARQ/ARQ	A	−	++	++	+++	+++	+	+	++	+++
R884 ARQ/AHQ	A	−	++	++	+++	+++	+	++	+++	+++
R882 VRQ/ARQ	A	−	++	++	+++	+++	+	++	++	++
R789 ARH/ARH	B	−	++	++	+++	+++	+	+	+++	+++
R816 VRQ/ARQ	C	+	+	++	+++	++	+/−	+++	+	+
R817 VRQ/ARQ	C	+	+	+	+++	+	−	+++	+	+
R790 VRQ/ARH	D	+	+	+	+++	+	−	+++	+	+
R646 VRQ/VRQ	E	+	++	+	+++	+/−	+/−	+++	+/−	++
R796 VRQ/VRQ	F	+	+	+	+++	+	−	+++	+/−	+
R617 ARR/ARR	G	−	−	−	−	−	−	−	−	−
R618 ARR/ARR	G	−	−	−	−	−	−	−	−	−
R647 ARQ/ARR	G	−	−	−	−	−	−	−	−	−
R682 ARQ/AHQ	G	−	−	−	−	−	−	−	−	−

* Electron microscopy lesions were ordered as negative, −; sparse, +/−; few, +; moderate, ++; widespread, +++; +ve, positive; −ve, negative.

After perfusion, brains were removed and two serial 1 mm slices were taken at the level of the obex. One of these slices was used for electron microscopy. The second slice and additional brain slices from representative brain areas were taken and processed into paraffin wax for PrP^d^ profiling (14, 15). Sections were mounted, dried overnight, dewaxed and immersed in 98% formic acid, then washed and autoclaved in 0.2% citric acid for 30 minutes. Endogenous peroxidase was inhibited with freshly prepared 3% hydrogen peroxide and slides were washed and blocked with 1:20 normal horse serum for 60 minutes. All slides were incubated with the rat monoclonal antibody R145. The wax-embedded medulla tissue sections were also immunolabeled using the technique described previously with the following antibodies: monoclonal anti-clathrin heavy chain (BD biosciences, Franklin Lakes, NJ, USA), monoclonal anti-clathrin light chain con1 (Sigma-Aldrich, Gillingham, UK), monoclonal anti-dynamin Hudy 1 (Upstate, Dundee, Scotland), monoclonal anti-dynamin D5 (Santa Cruz, York, UK), monoclonal anti-amphiphysin VAM-SV030 and VAM SV031 (Bioquote Ltd., Heidelberg, Germany), and polyclonal anti-ubiquitin (Dako UK Ltd., Ely, UK). Tissues were immunolabeled using these antibodies both with and without formic acid and autoclave steps.

Tissues for electron microscopy were selected under a dissecting microscope and embedded in araldite. Light microscopic labeling of 1-µm-thick plastic-embedded tissues for PrP was performed as described previously, but tissue sections were first etched using saturated sodium ethoxide immersed in 98% formic acid. Ubiquitin labeling was carried out as described previously; however, both labeling and section quality were improved with the omission of the formic acid step.

For electron microscopy, 55 nm sections were placed on 600-mesh gold grids and etched in sodium periodate for 60 minutes. Endogenous peroxidase was blocked and sections de-osmicated with 3% hydrogen peroxide in distilled water for 10 minutes followed by enhancement of antigen expression with 98% formic acid for 10 minutes. Primary antibody (523.7, a gift from Jan Langeveld) at a 1:250 dilution was then applied for 15 h at 27°C and incubated with Auroprobe™ 1 nm colloidal gold (Amersham Ltd., Amersham, UK). Sections were then postfixed with 2.5% gluteraldehyde in phosphate buffered saline (PBS) and labeling enhanced with Goldenhance® (Nanoprobes Ltd.) for 10 minutes. Grids were counterstained with uranyl acetate and lead citrate as described previously. In order to have exact correlation for individual cells and specific PrP^d^ accumulation types, serial 1-µm- and 55-nm-thick sections were labeled for PrP by light and electron microscopy, respectively. Ubiquitin labeling was carried out using Dako anti-ubiquitin® antibody at a 1:50 dilution without formic acid.

Sections stained for PrP^d^ were initially examined blind and the number of PrP^d^-positive structures were recorded as negative, sparse, few, moderate or marked. The immunogold labeling made identification of coated-pit associated changes more conspicuous, reflecting a greater magnitude of scoring, but the trends were identical to those reported by Ersdal *et al*(7) on the same material.

## RESULTS

### Light microscopy: two distinct pathological phenotypes indicate two strains

We assembled PrP^d^ profiles on each sheep as previously described (13, 15). Sheep could be divided into two groups according to the presence or absence of vascular plaques and astrocyte-related types of PrP^d^ accumulation (Table 1). Those in which plaques were absent tended to have higher total PrP^d^ scores (arbitrary units), principally manifested by higher amounts of stellate, perivascular and perivacuolar (white matter astrocyte-associated) PrP^d^ types. In contrast, sheep with vascular amyloid plaques had lower amounts of the glia-associated PrP^d^ types. When compared with previous data, these results show that two distinct separate strains or sources of scrapie were present in the 10 infected subjects.

**Figure 1:**
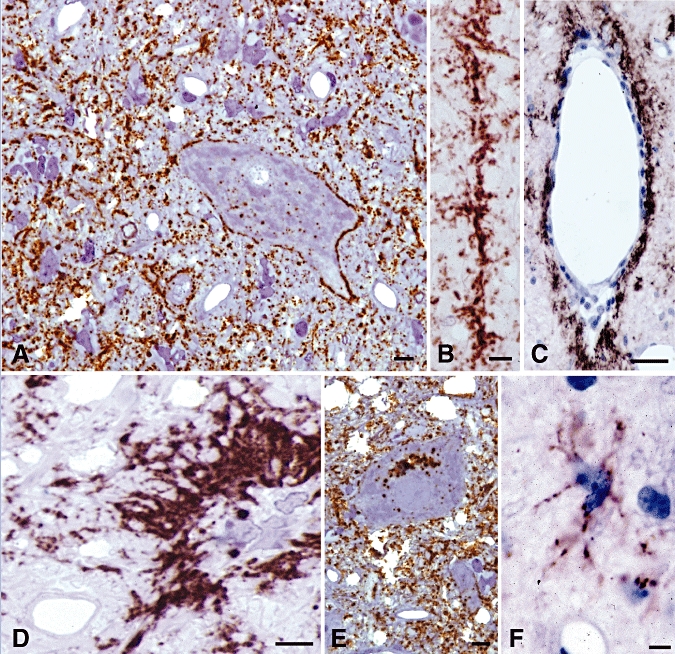
*Different types of PrP^d^ accumulation observed in 1-µm-thick immunolabeled sections*. In the neuropil of the dorsal motor nucleus of the vagal nerve (DMNV), most PrP^d^ accumulated in a diffuse punctate or granular pattern (A,E). Patterns of accumulation that clearly related to neurons included deposits around neuronal perikarya (A) and around neuronal processes as linear accumulations (B). PrP^d^ accumulation was detected within neuronal cytoplasm at all neuroanatomic sites as individual (A) and perinuclear puncta or clusters (E) of ovoidal granular deposits. Other patterns of PrP^d^ accumulation included perivascular (C) and intense coalescing patterns often seen around glial perikarya (D). Intense puncta of labeling, presumed to be intracellular, was often seen adjacent to glial cell nuclei (D). Though not distinguishable against the diffuse or granular neuropil labeling of the DMNV, a distinct pattern associated with the processes of astrocytes was seen elsewhere (F, olivary nucleus). Bar: A, 6 micron; B, 6 micron; C, 20 micron; D, 6 micron; E, 6 micron; F, 6 micron. Immunohistochemistry for PrP^d^ in 0.5–1-µm-thick plastic embedded sections using R523 antibody.

Three brainstem nuclei were selected for electron microscopic examination: the dorsal motor nucleus of the vagal nerve (DMNV), the spinal tract nucleus of the trigeminal nerve and olivary nuclei. Only some of the 13 possible types of PrP^d^ labeling present when whole sheep scrapie brains are examined (15) were present in these sites and are shown in Figure 1.

### Electron microscopy

#### Neuronal and dendritic plasmalemmal PrP^d^ is associated with abnormal endocytosis and cell membrane transfer

In previous studies of murine scrapie, we have shown that PrP^d^ is found mainly on the cell membranes of neurons and dendrites. The present study also shows that most sheep scrapie PrP^d^ is also found at the cell membranes of neurons and dendrites but not of axons. Cell membrane PrP^d^ was associated with abnormal endocytosis or was transferred to adjacent cell membranes. In contrast to murine scrapie, PrP^d^ was not aggregated into fibrils in the extracellular space.

To determine the relationship between light microscopic types of PrP^d^ and subcellular sites of PrP^d^ accumulation, individual patterns were identified by light microscopy in 1-µm-thick immunolabeled sections and then located in serial sections by electron microscopy. The light microscopy patterns of perineuronal, linear and diffuse punctate labeling (Figure 1A,B,E) all correlated at electron microscopy with PrP^d^ located at perikaryonal and dendritic plasma membranes.

**Figure 2:**
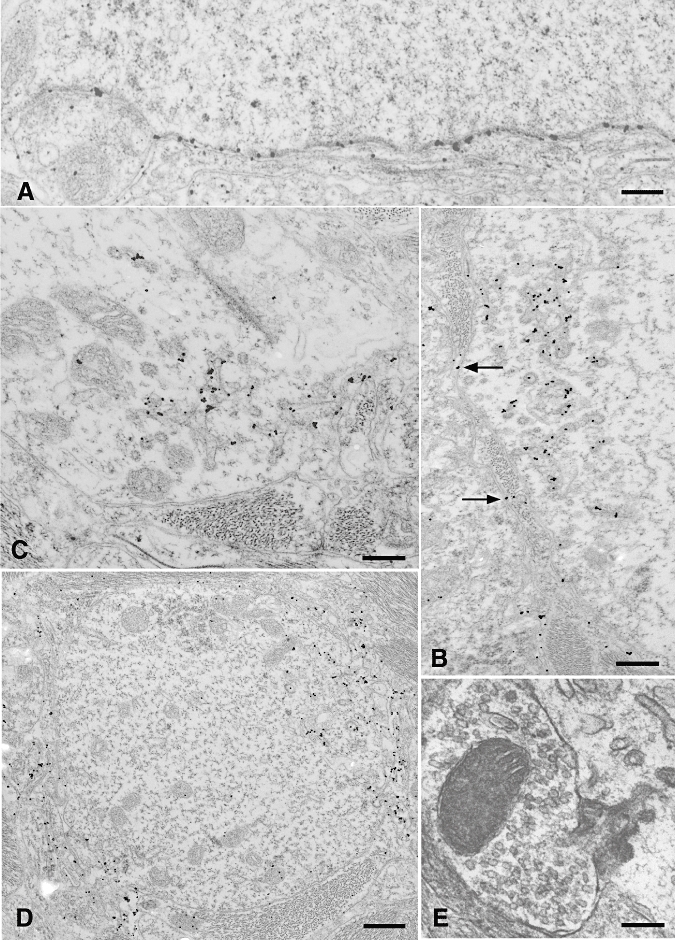
*PrP^d^ accumulation was found at the surface of neurons and dendrites corresponding to perineuronal, linear and punctate patterns of labeling at light microscopy.* Some PrP^d^ accumulation occurred at the plasma membrane of neurons and dendrites in the absence of any other changes (A). Some membrane accumulation was associated with the formation of excess coated pits and with the transfer to membranes of adjacent processes (B). In appropriate sections, the continuity between coated pits with abnormally long necks and the cell surface could be established (B,C,E). Some of these pits formed fused tubular networks beneath the cell membrane (B,C). In dendrites, PrP^d^ often accumulated at two poles of the same process, and with increasing accumulation PrP^d^, could be detected on membranes adjacent to the cell (D) (see also arrows at B). Complex dendrite membrane disturbances associated with the formation of abnormal pits incorporated the membranes of adjacent processes; these were usually astrocytic, but rarely, axon terminals were also involved (E). Bar: A, 0.29 micron; B, 0.39 micron; C, 0.30 micron; D, 0.53 micron; E, 0.19 micron. A–D, PrP^d^ immunolabeling using R523 antibody; E, uranyl acetate/lead citrate, not immunolabeled.

In relation to each of the previously mentioned light microscopic types of PrP^d^ labeling, including the punctate patterns, some PrP^d^ was located precisely at the plasmalemma of neuronal cell bodies and of dendrites with no other changes evident, neither of the adjacent neuropil nor of the neuronal cell body or dendritic cytoplasm (Figure 2A).

A fraction of neuronal and dendritic plasmalemmal PrP^d^ was associated with morphological changes. PrP^d^ was associated with numerous abnormal coated pits and vesicles (Figure 2B,C). In appropriate planes of section, vesicles were connected to the cell surface by tubular, sometimes spiral, invaginations of the cell membrane. PrP^d^ labeling was found within these connecting twisted tubules and in the associated coated structures and pits (Figure 2B,C). Fusion of pits and vesicles resulted in complex subplasmalemmal tubular networks (Figure 2B,C). Often, pits and associated changes were present at two poles of a cross-sectioned dendrite (Figure 2D); and in longitudinal sections of dendrites, they were present as discrete focal areas distributed discontinuously along a process. Where the entrance to the coated pits was relatively broad, adjacent processes, usually astrocytic but occasionally dendritic, and rarely, axon terminal membranes (Figure 2E), extended into the mouth of the open pits. Close proximity, and rarely, continuity between coated membranes and PrP^d^-positive endosomes or lysosomes was occasionally seen. Similar structures were not found on axons.

PrP^d^ was also seen on the plasmalemmas of processes adjacent to PrP^d^-positive dendrites and neuronal soma (Figure 2D). The labeling of adjacent processes was indiscriminate and could involve astrocytic processes, axonal structures, occasionally including synaptic boutons (though we never observed involvement of synaptic densities), as well as other dendrites. The surface contours of the dendrite could be very convoluted, some showing finger-like cytoplasmic extensions or microfolds of the plasmalemma (Figure 2D). The extracellular space between these folded membranes was often increased.

#### Astrocyte-associated PrP^d^ is mainly related to plasmalemmal microfolding

**Figure 3:**
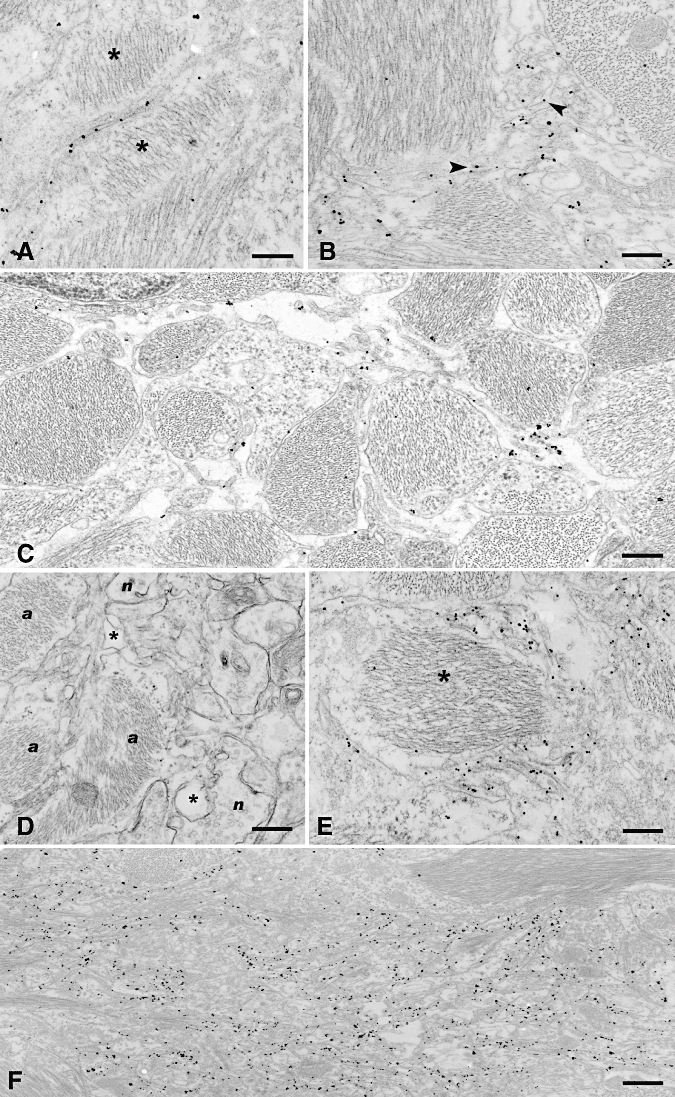
*Patterns of accumulation of PrP^d^ in association with astrocytic plasmalemma*. As for dendrites, some PrP^d^ accumulation was seen at the plasmalemma with little other change present, but most PrP^d^ was associated with a proliferative membranous change. The simplest pattern of PrP^d^ labeling was present at the plasmalemma as seen between two adjacent astrocytic (stars) processes in (A). In (B), PrP^d^ labeling is seen on the plasma membrane of small cytoplasmic projections (small arrows) arising from astrocytic processes containing abundant glial filaments. In areas where astrocytes form a dense network of processes, the nature of the microfolding of these projections can be more clearly visualized (C) and is seen as PrP^d^-labeled filiform cytoplasmic projections originating from and running between glial processes. The complex nature of the membranes around astrocytes is shown in a nonimmunolabeled section in (D); a indicates astrocytic processes, n neuronal processes and star expanded extracellular space and in a PrP^d^-labeled section in (E). The association of PrP^d^ with these convoluted membranes is shown around an astrocyte process containing a large bundle of intermediate filaments (star). The coalescing pattern around glial cells such as shown in Figure 1D translates to an area comprising extensive PrP^d^-labeled membrane microfolds and convolutions between glial bundle-containing astrocytic processes (F). Bar: A, 0.27 micron; B, 0.30 micron; C, 0.32 micron; D, 0.44 micron; E, 0.25 micron; F, 0.60 micron. A–C,E,F, PrP^d^ immunolabeling using R523 antibody; D, uranyl acetate/lead citrate, not immunolabeled.

When located in serial light and electron microscope preparations, perivascular, coalescing and periastrocytic types of PrP^d^ accumulation (Figure 1C,D,F) were associated with the plasma membrane of astrocytes (Figure 3A). Even presumed early accumulations of PrP^d^ at the plasma membrane of astrocytes were associated with complex membrane changes (Figure 3B–E). In the presence of PrP^d^ at the cell surface, astrocytic process plasmalemmas became markedly irregular with many small microfolds or projections. In areas such as the adjacent reticular formation or the subependymal neuropil which contained closely packed astrocytes, the nature of these finger-like projections were more obvious (Figure 3C). In other areas, the membranes had a linear form, which on immunolabeling were strongly positive for PrP^d^. Where PrP^d^ accumulations associated with astrocytes were intense, astrocyte processes were characterized by prominent glial bundles, a markedly disordered neuropil and numerous microglial processes. Such areas correlated with a coalescing pattern of PrP^d^ accumulation at light microscopy (Figure 3F). Rare PrP^d^-labeled coated pits were seen but not in association with abnormal membrane invaginations.

#### Intracellular PrP^d^ labeling is located in lysosomes

**Figure 4:**
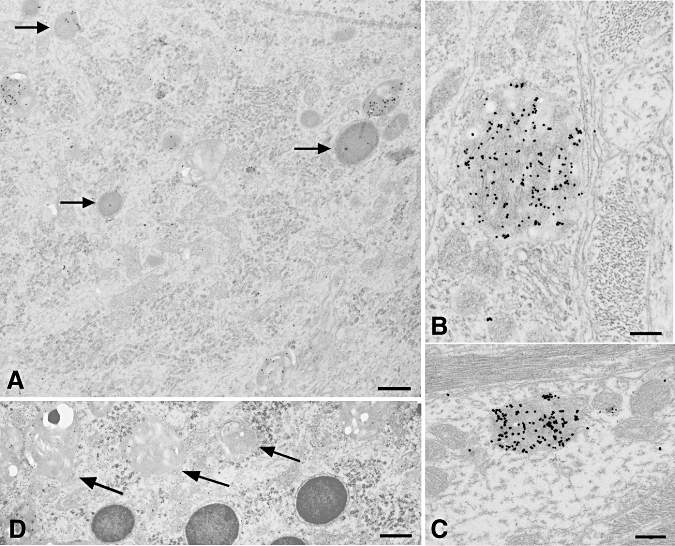
*Intralysosomal PrP^d^ accumulation was found in several cell types*. In neurons, levels of labeling were higher on immature lysosomes, identified by a less electron dense content, than on mature lysosomes (arrows) (A). Intralysosomal PrP^d^ was also identified in astrocytic and microglial glial cell bodies and processes (B) and in dendrites (C). Immature lysosomes of neurons were generally paler, often vacuolated and irregular in shape (D). Bar: A, 0.88 micron; B, 0.29 micron; C, 0.33 micron; D, 0.81 micron; A–D, PrP^d^ immunolabeling using R523 antibody.

At light microscopy, intracellular labeling was evident in neurons (Figure 1A,E), astrocytes (Figure 1D) and microglia. Electron microscopy showed that PrP^d^ was located within mature and immature lysosomes of neuronal perikarya (Figure 4A,D), occasional dendrites (Figure 4C) and glial cells (Figure 4B). In favorable planes of sections, continuity between subplasmalemmal endosomal or lysosomal structures and abnormal pits invaginating from the cell membrane could be identified, suggesting intra-endosomal and intralysosomal PrP^d^ was derived from the cell surface PrP^d^.

Many neurons contained very frequent lysosomes and endosomal–lysosomal structures, particularly in the perinuclear Golgi zones. To determine whether lysosomes were increased in these sheep, we performed cathepsin immunolabeling at light microscopy and counted endosomal-lysosomal structures by electron microscopy. No increased light microscopic labeling for cathepsin was observed, but substantially more endosomes–lysosomes were encountered in neuronal perikarya of scrapie-infected neurons when compared with controls in formal morphometric examination (data not shown).

#### PrP^d^ is associated with only some scrapie-specific cytopathological changes

As is common with chronic end-stage neurological disease, many different lesions are present in brains of clinically sick scrapie-infected animals. Some of these changes are nonspecific, but a number of changes are pathognomonic or highly indicative of scrapie infection. As partly described previously, some scrapie-specific lesions were closely associated with PrP^d^ but other changes were not.

Several morphological features have been described in TSE-affected brains that we consider to be pathognomonic. These are tubulovesicular bodies (31), spiral membrane inclusions of axons previously referred to as spiroplasma (1), highly abnormal coated pits (7) and membrane proliferation or microfolding. Vacuolation is not itself pathognomonic at light microscopy, but the nature of tissue changes that surround and are present within vacuoles appears to be a type of spongiform change unique to the TSEs.

As in previous studies of murine scrapie, the membrane and contents of vacuoles and tubulovesicular bodies were unlabeled (20, 32). None of the major nonspecific degenerative features observed, including cerebellar lamellar bodies (8), clumped synaptic vesicles (2), degenerate processes, dystrophic axons or autophagic vacuoles, were labeled.

Spiral membrane inclusions occurred predominantly in axons and synaptic boutons. Continuity with the cell surface was occasionally evident. Unlike dendritic and neuronal membrane invaginations, the coating of the axonal spiral inclusions was electron lucent and lacked buds or branches. Some PrP^d^ labeling was present but to a much more limited extent than that found on the abnormal coated pits. We hypothesize that these forms are specific, predominantly axonal responses to dendrite-derived PrP^d^ at the axonal plasma-membrane.

#### Cell membrane PrP^d^ is endocytosed via a ubiquitinated classical pathway

Clathrin-coated pits are involved in the endocytosis and recycling of cell membrane proteins including normal PrP^c^(44). The bizarre extensions and branching of the necks of coated pits led us to consider the possibility that molecular abnormalities of the endocytosis and recycling of PrP^d^ might be involved in these changes. We therefore investigated proteins involved in the formation of coated pits and in their excision from the plasma membrane in several light microscopic studies. Immunohistochemistry of clathrin, dynamin and amphiphysin showed no significant changes (data not shown). However, ubiquitin, which is involved in tagging proteins for proteolysis in endosomal-lysosomes was increased.

**Figure 5:**
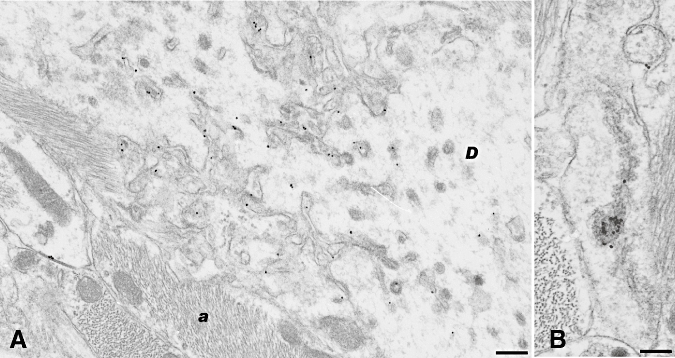
*Ubiquitin labeling*. Conspicuous ubiquitin labeling was present on abnormal coated pits at the cell membrane of dendrites (D) and on coated pits and vesicles and on the subplasmalemmal fused tubular complexes. It was also present on membranes of cell processes [in this case astrocytic processes (A)] adjacent to such areas. Variable; sometimes high levels of ubiquitin labeling were found on multivesicular bodies and on endosomal-lysosomal structures, shown here attached to spiral inclusions (B). Bar: A, 0.37 micron; B, 0.24 micron; immunogold for ubiquitin.

By light microscopy, punctate ubiquitin immunolabeling was present in neuropil of varying intensity in scrapie-affected sheep but not in controls. For electron microscopy, paired thin sections were labeled for PrP^d^ or ubiquitin to confirm sites of PrP^d^ accumulation on individual sections and then to determine levels of ubiquitin at these and other sites. Conspicuous ubiquitin labeling was associated with abnormal coated pits and subplasmalemmal tubular networks wherever these occurred on dendrites or neuronal cell bodies (Figure 5A). Ubiquitin was also present on the membranes of other cell processes immediately adjacent to these structures (Figure 5A). In paired sections, the PrP^d^ labeling was present in corresponding locations. Some multivesicular bodies in dendrites were labeled strongly for ubiquitin in both controls and scrapie-affected sheep, though in the latter, these structures were either not labeled for PrP^d^ or showed only slight labeling. Weak labeling for ubiquitin was found on endosomal–lysosomal structures in neuronal perikarya, and rarely, in endosomal-like structures linked with the spiral membrane inclusions (Figure 5D).

#### Effect of scrapie strain on subcellular lesions and PrP^d^ accumulation

The same range of morphological features and immunolabeling was seen in each of the sheep examined, but the frequency of changes differed markedly between individuals. When sheep were allocated to strain type, two distinct groups were evident. Thus, the two scrapie strains present differed according to discrete differences in the proportions of lesions present (Table 1).

At the level of the DMNV or olivary nuclei, there were few differences in the light microscopic patterns of PrP^d^ accumulation when each of the 10 scrapie-affected sheep was examined. A semiquantitative analysis of electron microscopy lesions present in the DMNV and in olivary nuclei was performed on coded grids. When the magnitude of selected features are tabulated (Table 1), the 10 scrapie-affected sheep can be divided into two distinct groups (Table 1) that correspond with the strains identified by light microscopic PrP^d^ profiling. The first group showed marked formation of PrP^d^-associated abnormal coated pits and associated membrane irregularity and invaginations, increased lysosomes in neurons (mostly PrP^d^ positive) and had more readily detectable spiral membrane inclusions. The five remaining sheep also had each of these features but in much less abundance. However, this second group of sheep had marked PrP^d^-associated proliferative and microfolding membrane changes in astrocytes. These changes are morphologically distinct from classical reactive astrocytosis. Thus, when the light microscopy strain typing approaches and the electron microscopy changes are considered together, the same groupings are present and show that interanimal differences in PrP^d^-related pathological changes differ only in magnitude and not in nature. Nevertheless, these magnitude changes segregate according to the strain of infection.

## DISCUSSION

The present study informs on the *in vivo* cellular and subcellular location of PrP^d^ and its relationships to subcellular morphological changes. From these observations, we can infer the sites of conversion of PrP^c^ to PrP^d^ and the inter and intracellular trafficking of PrP^d^ in two strains of sheep scrapie.

### PrP^c^ to PrP^d^ conversion occurs at the cell membrane

In agreement with conventional murine scrapie models that show lysosomal, cell membrane and extracellular PrP^d^ accumulations (20, 21, 22), this study shows that PrP^d^ of scrapie-affected sheep is also associated with endosomes, lysosomes and plasma membranes of neurons and glia. In previous studies of sheep scrapie and experimental ovine BSE, we have shown that intracellular PrP^d^ accumulations are N-terminally truncated (23, 24), but all other neuropil types of PrP^d^ accumulations are full length. When compared with these data, the present results suggest that PrP^d^ first accumulates at the cell surface of neurons and dendrites from where it is either released or internalized and then digested. *In vitro* pulse-chase experiments first suggested that the transformation of normal to abnormal PrP probably takes place at the cell surface or at some later stage in the cell cycle (5). Our results therefore agree with this data insofar as the first site of the cell cycle at which inferred full-length PrP^d^ is visualized is at the cell membrane.

### PrP^d^ is internalized by an abnormal classical endocytotic mechanism

Consistent with other glycosylphosphatidylinositol (GPI)-anchored proteins, some studies suggest that PrP^c^ is internalized by one of the noncoated pit mechanisms for raft-associated proteins (27, 39), but others show that PrP^c^ is internalized from N2a cells via coated pits (42). In N2a cells, PrP^c^ resides within the lipid-rich fragments of the membrane but leaves these regions to be endocytosed within nonraft regions by a clathrin-coated mechanism (44). As GPI-anchored proteins do not have a transmembrane domain, they cannot directly interact with clathrin-associated proteins to elicit endocytotic vesicle formation; basic residues within the N terminus of PrP^c^ are necessary to link between it and other membrane ligands that possess cytoplasmic domains recognized by clathrin (44). The present study suggests that PrP^d^ on neuronal cell membranes is also internalized via a coated-pit mechanism.

The bizarre tubules connecting coated pits to the cell surface suggest that the excision of the PrP^d^-containing coated pits from the plasma membrane of scrapie-infected neurons occurs inefficiently. We hypothesized that PrP^d^ might interfere with the excision of pits from the cell surface and attempted to identify potential molecular defects using antibodies directed at proteins involved in vesicle excision, but we were unable to find any consistent changes in the abundance of amphiphysin, dynamin or clathrin (data not shown). We therefore propose that PrP^d^ alters endocytosis by interfering with one of the vesicle excision proteins, which then elicits excess ubiquitin amplification prior to endosomal degradation of such complexes. Similar features are found in the scrapie-infected TG3 PrP^0/0^ mouse, which generates PrP^d^ from astrocytes and lack neuronal PrP^c^(25). This suggests that astrocyte-derived PrP^d^ can be transferred to neuronal membranes and drive abnormalities of endocytosis of abnormal PrP^d^. Abnormal endocytosis may therefore be an acquired toxic change of PrP^d^ and may not require any alteration in cytoplasmic expression of proteins associated with clathrin-mediated endocytosis. In the present study, astrocytes of scrapie-infected sheep showed PrP^d^ internalization by normal coated pits. We have also shown, in lymphoid tissues of the same sheep described here, that abnormal endocytotic mechanisms are present in macrophages internalizing PrP^d^. However, the macrophages internalize the PrP^d^ via a noncoated (probably caveolin) mediated mechanism (36). Together, these observations suggest that membrane processing of PrP^d^ is independent of PrP^c^ expression and is influenced by cell type.

Scrapie-associated alterations of the ubiquitin–proteosomal system are reported (6, 28, 47), with one study proposing that neurodegeneration may be caused by abnormally trafficked PrP^c^(34). In these scrapie-infected sheep, PrP^d^ and ubiquitin were present together at the cell surface, in abnormal coated pits, in fused subplasmalemmal tubular networks and in a proportion of lysosomes but not in association with the endoplasmic reticulum or the cytosol adjacent to Nissl or Golgi bodies, suggesting that ubiquitin-associated trafficking of PrP^d^ does not involve the proteosome. Rather, these results suggest that PrP^c^ conversion or PrP^d^ accumulation signals to a cytosolic protein that can instigate the clathrin endocytotic process, either directly—as a consequence of an abnormal location within the membrane—or more probably by complexing with another ligand as previously suggested (42). This protein complex is not recycled but directed to the endosomal system for degradation by ubiquitin signaling, which appears to increase cellular catabolic activity as shown by increased lysosomes. Astrocyte membrane-associated PrP^d^ was not detectably endocytosed with ubiquitin. Thus, mechanisms by which astrocytes and neurons deal with cell membrane-associated PrP^d^ are different.

### Intercellular transfer of PrP^d^

Scrapie-infected cells *in vitro* demonstrate release of PrP^d^ and/or infectivity into the culture medium (9), and we have previously shown that PrP^d^ may also be released from the surface of scrapie-infected mouse neurons and dendrites *in vivo*(21). Exosomes are 50–90-nm-diameter vesicles of endosomal origins that are released into the extracellular environment and can transfer PrP^c^ from neuroblastoma cells to PrP null cell lines (33). We were not able to identify exosome-like structures in routine preparations in this or in previous rodent studies. To eliminate the possibility that small numbers of exosomes might by chance escape detection in two-dimensional membrane representations seen at electron microscopy, we performed serial reconstructions of putative PrP^d^-containing membrane fragments but failed to detect any exosomes (data not shown).

Previous studies have shown that GPI-anchored proteins, including PrP, may be transferred between membranes, sometimes as a complex of molecules (33, 19). Our observations suggest that a fraction of PrP^d^ that reaches the cell membrane is then transferred to other membranes of adjacent processes. This transfer is indiscriminate and independent of cell type and also occurs in the lymphoid tissues of these sheep (36). As the TG3 PrP^0/0^ mouse also demonstrates PrP^d^-associated pathology on PrP null neurons (25), we can also infer that the transfer of PrP^d^ between cells is independent of PrP^c^ expression. Because both ubiquitin and PrP^d^ are seen on membranes adjacent to a scrapie-infected PrP^d^-releasing cell, we surmise that PrP^d^ is moved between membranes as a multimeric protein complex.

### PrP^d^ largely accumulates on dendritic rather than axonal membranes

Most brain PrP^c^ appears to be located in the neuropil (10), and previous light microscopic studies have sought to relate the diffuse neuropil accumulation of PrP^d^ to synapses (29). Some studies have suggested a preferential synaptic membrane concentration of PrP^c^(11, 38), but others show generalized localization on plasmalemmas (16, 30, 36). In these latter studies, PrP^c^ was found to follow standard biosynthetic pathways for GPI-anchored proteins, with PrP^c^ expressed in Golgi bodies and recycled through late endosomes after reaching the cell membrane.

At the cell surface, the GPI anchor and N-terminal domains direct PrP^c^ to detergent-resistant membrane (DRM) fractions (45). However, when the DRM lipid content is experimentally altered, PrP^c^ is redistributed from axons to dendrites (12). In the present study, as in previous studies of murine scrapie, we show that PrP^d^ accumulations in sheep are primarily related not to axons or to synapses but to dendrites. The observed preference for PrP^d^ accumulation and release from dendrites may therefore be related to altered trafficking of the abnormal protein within the membrane or to an altered distribution of PrP^c^ and PrP^d^ following changes in the raft domains of axonal and dendritic membranes.

### Scrapie strains or sources differ in the proportion of functional PrP^d^ conformers

When the 10 scrapie-infected sheep investigated in the present study were segregated according to strain or by their electron microscopy cytopathology features (Table 1), they segregate into the same two groups. The results therefore suggest that there are strain-directed cytopathological effects of scrapie infection. However, the differences in cytopathological changes observed between the two strain groups were not qualitative but quantitative; that is, the same PrP^d^-associated ultrastructural pathology was recognized for the two strains but in different proportions. This is in agreement with previous light microscopic immunohistochemical observations, suggesting that different strains may target some cell populations rather than others, giving rise to, for example, a more prominent neuron-associated rather than glia-associated morphological pattern of PrP^d^ deposition, and that infection with different strains may lead to a different processing of PrP^d^, which is translated into, for example, more abundant intracellular rather than cell membrane aggregates (14).

Different cell types, such as neurons, glial cells and follicular dendritic cells (36), can exhibit different cytopathological changes when infected with the same strain of TSE agent; this is most likely caused by biological differences between these cell types. However, diversity of ultrastructural morphological changes can also be found when a single strain of TSE agent infects a particular cell type. Thus, a neuron can internalize cell membrane PrP^d^ via abnormal coated pits, show PrP^d^-associated plasma membrane microfolds and can release membrane PrP^d^ to the extracellular space or transfer it to the membranes of adjacent cells. These different processing mechanisms result in the diversity of ultrastructural changes described, and the fact that these vary quantitatively, not qualitatively, in infections by different strains can be interpreted as follows: PrP^d^ originating from infection with one particular strain interacts mainly with one cell membrane protein resulting in a main processing pathway and pathological change, and less so with other cell membrane proteins, resulting in different, secondary trafficking pathways and less pronounced cytopathology features. In this scenario, PrP^d^ produced following infections by different strains would have distinct relative affinities for different cell membrane proteins, resulting in diverse proportions of processing mechanisms and pathological changes. It is conceivable that for this to happen, PrP^d^ from a single strain would consist of several tertiary or quaternary isoforms, each of which would interact with a different cell membrane protein; different proportions of those isoforms in different TSE strains would result in relatively diverse trafficking mechanisms and different proportions of essentially the same cytopathological changes, as observed. Either through interaction with other cell membrane proteins or on its own, this notion of TSE strains being composed of several PrP^d^ isoforms in varying proportions—rather than consisting solely of single isoforms—appears to fit better with the consistency of pathological changes observed for particular strains. If prion strains generated only single isoforms, the diversity of processing mechanisms and the different resulting cytopathological changes would be best explained as random events that would not lead to consistent pathological phenotypes for different strains.

The scrapie-infected TG3 PrP^0/0^ mouse shows the same PrP^d^-associated neuronal coated pit and membrane changes (25) as found in sheep. Despite the astrocytic origin of infection in this transgenic model, these mice did not show the same level of astrocytic plasma membrane proliferation of the sheep. This indicates that the responses of both astrocytes and neurons are also directed by specific properties of PrP^d^ and not simply by the presence of infectivity in different cell types. It further suggests that it is the nature of the PrP^d^ isoforms that dictates the abnormal cytopathology and inferred trafficking and not the converse. In general, rodent scrapie infections release more PrP^d^ to the extracellular space and internalize less than sheep sources; nevertheless, the nature of the tissue changes and sites of PrP^d^ localization are similar for rodent and sheep scrapie strains. We therefore suggest that every TSE or prion infection produces a range of PrP^d^ isoforms, each of which produces different cytopathological effects. The relationship between different forms of PrP^d^ and infectivity, and the significance of certain prion disease-specific, PrP-negative cytological changes such as tubulovesicular structures have yet to be established.
